# Medical specialty certification in the United States—a false idol?

**DOI:** 10.1007/s10840-016-0119-4

**Published:** 2016-03-08

**Authors:** Westby G. Fisher, Edward J. Schloss

**Affiliations:** 1 NorthShore University HealthSystem, Evanston, IL USA; 2 Pritzker School of Medicine, University of Chicago, Chicago, IL USA; 3 The Christ Hospital, Cincinnati, OH USA

**Keywords:** Medicine, Certification, Specialty, Physician, Re-certification, Ethics, Medical education, Health policy

## Abstract

**Purpose:**

Recent changes to medical specialty certification in the USA have prompted the process to come under intense scrutiny.

**Methods:**

We review the history of board certification and the changes made to the process. As part of this review, we examine both literature and public record to examine the motives behind the changes made. We then review the legal challenges and changes under way to modify the current ABMS board re-certification process.

**Results:**

In 1917, the first board certification was a lifetime designation, voluntary, and managed by unpaid board members with a focus to enhance quality for patients. Corresponding to the implementation of time-limited certification, $55 million of physician testing fees were transferred from the American Board of Internal Medicine to its Foundation between 1989 and 1999. From 2000 through 2007, and additional $20.66 million were transferred from the ABIM to its Foundation culminating in the purchase of a $2.3 million luxury condominium in December 2007.

**Conclusions:**

Significant financial conflicts of interest for the implementation of time-limited specialty certification exited and continue to plague the medical profession. The specialty boards and the organizations that created them should remove all requirements for time-limited board certification and resort to conventional self-selected ACGME-approved CME programs for ongoing education.

“A profession is a vocation founded upon specialized educational training, the purpose of which is to supply disinterested objective counsel and service to others, for a direct and definite compensation, wholly apart from expectation of other business gain.” [1]

Medical practice in the USA today is structured around physician-specialists. Training and credentialing of these specialists is essentially unregulated by government. Instead, a comprehensive private, self-appointed regulatory system has been developed, based largely on standards created by 24 independent, tax-exempt corporations called “specialty boards” of the American Board of Medical Specialties (ABMS). Collectively, $374 million from US physicians funded these specialty board organizations in 2011 [2]. Recent events have led some in the medical community to question the legitimacy of these specialty boards to uphold the interests of practicing physicians and their patients. This paper will review the facts that have led to the controversy and review solutions that are underway to improve the transparency and integrity of the specialty certification system in the USA.

## The history of the specialty board: a quantitative quest for medical education adequacy

At the beginning of the twentieth century, American medical education was poorly standardized. Medical education requirements, training, and assessment of clinical competence varied widely among medical schools. As the number of those practicing medicine grew, state legislatures took up the issue of physician licensure. At the same time, physicians associations were forming in states and larger cities with the principle focus on improving medical care. They also saw their responsibility to work for issues of benefit to physicians. Therefore, the classic conflict of interest for professional groups was already evident, “that professionals and the professions act with a dual motive: to provide service and to use their knowledge for economic gain” [3]. Despite these efforts, much of medical care remained of low quality, in part because educational standards in medicine were virtually non-existent.

In 1904, the American Medical Association created the Council of Medical Education (CME) with the aim of restructuring American medical education [4]. The CME proposed a set of basic standards for medical school admission and training. In 1908, the CME asked the Carnegie Foundation for the Advancement for Teaching (which had been chartered in 1906 by an act of Congress) to survey American medical education, so as to promote the CME’s reformist agenda and hasten the elimination of medical schools that failed to meet the CME’s standards. The president of the Carnegie Foundation chose Abraham Flexner to conduct a survey of all 155 US medical schools at the time. He published his findings and recommendations for medical school reform in the now infamous Flexner Report [5]. This report set the framework for our current medical education requirements. Ultimately, only 66 medical schools remained open and able to meet the standards for medical education proposed by Flexner and the CME. Effective education was recognized as the key to quality.

At the time of the Flexner report, the remaining US medical schools generally shunned the concept of medical specialization [6]. The rapid rise in scientific knowledge about diseases and treatment, population growth that allowed for a concentration of patients with similar diseases, and the development of new skills and techniques among those practicing in narrower fields of practice contributed to the rise of medical specialization [7].

## The first specialty board

In ophthalmology, the improved understanding of refraction had profoundly changed ophthalmic practice so that an apparently menial task originally left to opticians and jewelers became of economic importance to oculists. Early in the 1900s, oculists were organizing themselves as optometrists and were seeking state licensure to perform refraction and other tasks. Optometry quickly emerged as a threat to ophthalmology and efforts were stepped up to block its acceptance in state legislatures [7]. Ophthalmologists realized that specialized education was urgently needed, as well as some means of differentiating those who met standards of competence through quality education from others who were less well trained. The innovative concept of the “specialty board” to oversee training and evaluation of physicians who desired to specialize in ophthalmology following medical school began to crystalize. By 1915, a joint committee of ophthalmologic societies including the AMA drafted a report recommending the establishment of a board “to arrange, control, and supervise examinations, to test the preparation of those who design to enter on the special or exclusive practice of ophthalmology.” In 1917, the first specialty board in medicine, the American Board for Ophthalmic Examinations, was incorporated and its name was later changed to the American Board of Ophthalmology in 1933 [8]. Standards for specialty boards at the time required no government affiliation, no influence on the ability of physicians to practice their trade, and board members were to be unpaid. “It was recognized that an authoritarian approach to board certification, coupled with the threat of loss of licensure and loss of income, was unlikely to be acceptable in the egalitarian United States” [7].

Other medical specialty organizations quickly followed suit. In 1933, the American Board of Medical Specialties (ABMS) was incorporated stemming from a request by the AMA. Three years later, the American Board of Internal Medicine (ABIM) was incorporated in 1936 in Des Moines, Iowa, and quickly grew to the largest specialty board regulating 12 internal medicine subspecialties.

From 1941 until 1989 after graduation from medical school and doing defined years of specialty study, physicians would sit for an initial specialty board examination in their area of expertise administered by their specialty board. Passing the examination was sufficient for board certification for the rest of their career. In December of 1986, “after more than 20 years of discussion and debate,” the American Board of Internal Medicine elected to join 17 other specialty boards by limiting the duration of validity for all certificates it issued [9]. The rationale for this change was that “medical information changes rapidly and the public needs reassurance that certified internists have maintained their skills and kept their knowledge up to date” and “evidence that knowledge and skills of practicing decay with time (albeit at variable rates)” [10]. The ABIM also published that time-limited certification and re-certification are “obligations of an accountable profession” [11]. Several attempts at providing voluntary re-certification programs for board certificate holders were met with “only limited and declining interest” [9].

## The new certification era: “asking, but not requiring” renewal of certification

Faced with the reality that practicing physicians were unenthusiastic about re-certification, the stage was set for the ABIM to issue an ultimatum: “diplomats would be asked, but not required, to renew the validity of the certificates at periodic intervals or face the uncertain consequences of loss of their status as certified internists, subspecialists or holders of certificates of added qualifications” [9]. This watershed moment forever changed the landscape of specialty certification from one that primarily served the needs of practicing physicians to one that threatened “uncertain consequences” and mandated additional requirements designed in large part to serve the ethical views and ongoing financial needs of the Specialty Boards. The first implementation of this policy decision resulted in the deployment of the ABIM’s Continuous Professional Development (CPD) program. This first internal medicine re-certification examination under the new time-limited CPD certification program was administered in 1996 to 256 participants sitting for 306 examinations in 12 subspecialty areas with a 93 % overall pass rate [12]. The following year, 867 candidates registered for CPD re-certification [12].

A relatively short time later in 2005, more requirements were added by the ABMS for re-certification and the re-certification program rebranded as “Maintenance of Certification” (MOC). With MOC, a certain number of “MOC points” had to be accrued by various performance improvement projects and data collection exercises before a physician could sit for his re-certification examination. Then in 2014, the ABIM doubled the MOC points required (from 50 to 100 every 5 years) and stipulated that physicians who did not meet the requirements of this expanded program would be publicly labeled on a sponsored website as “not meeting MOC requirements.” These changes occurred with little public commentary and at a time when physicians’ clinical time was already stressed by implementation of the electronic medical record, the transition of many from private practice to employed status with larger “health care organizations,” increased quality reporting requirements, and new billing and compliance requirements. These new re-certification mandates were conceived or overseen by ABMS-imposed leadership officers of whom only 9 % collectively had recertified in general medicine and 25 % had recertified in any certified subspecialty [13].

## Current state of specialty board certification—the proficiency conundrum

Today, board certification of medical specialists is considered voluntary and is not legally required in order to practice medicine in any jurisdiction [14]. Likewise, neither the boards nor any other medical organization encourages health care institutions to limit specialty practice to certified physicians alone [14]. These realities notwithstanding, certification profoundly affects physicians’ professional opportunities. Since more than 90 % of US medical school graduates complete a period of post-graduate training in the form of “residency,” the broad acceptance of post-graduates to become “board certified” gives initial certification significant impact with hospital privileges, peer and patient recognition, economic compensation, and the standard of care [14]. The change of permanent board certification to time-limited certification in 1990 effectively converted the “voluntary” aspect of board certification to a requirement to maintain hospital privileges and insurance panel participation and profoundly impacts a physician’s ability to earn a living in the event of re-certification failure. Despite this reality, to the best of our knowledge, no study has ever been conducted examining the psychological, economic, and employment outcomes of physicians who fail one or more re-certification examinations.

## The skyrocketing costs of maintenance of certification

The cost of participating in MOC in general medicine mushroomed 244 % (or 16.3 % per year) from $795 in 2000 [15] to $1940 in 2014 [16]. Similarly, the cost for subspecialty re-certification grew 257 % (or 17.2 % per year) over the same time period. A recent cost analysis estimated general internists incur an average cost of $23,607 (95 % CI $5380 to $66,383) and cardiac electrophysiologists incur an average cost of $52,196 (95 % CI $9773 to $115,916) in total MOC costs over 10 years [17]. The view by many practicing physicians at the time was this re-certification program did not make them better physicians and represented poor value for their money with unnecessary paperwork and data entry exercises. Worse yet: many saw themselves as cramming a vast array of trivia in their heads just to stay employed since hospitals nationwide were increasingly incorporating time-limited “maintenance” of board certification into their credentialing policies [18]. Nearly 23,000 physicians signed a petition to revoke the changes made to the MOC program [19].

No ethical practicing physician argues with the need to remain proficient given the rapidly changing innovations in our field. In fact, physicians deal with the rapid growth of medical information and innovation on a daily basis. Thanks to dramatic technological advances in information systems and development of the worldwide web, information is available to both physicians and patients nearly instantaneously. A physician’s training and experience in assessing the source and evidence base for any new medical development allows them to critically assess its applicability and appropriateness for patients while respecting the legal liability inherent to incorrect judgments in this regard. So integral and commonplace are these skills for medical practice that state licensure boards have always required proof of this commitment to continuing medical education (CME) through the accumulation of a minimum of CME credits every several years with no serious legal judgments filed before a physician’s state license is renewed. In this respect, the MOC re-certification program adds little more than an additional burden to physicians’ time and finances.

Proponents of the MOC program say its goal is to “reassure the public that the practitioner continues professional development and education post-training” [8]. There would have to be compelling evidence in order to legitimize a re-certification process that has become a prerequisite for hospital credentials [16], time-consuming and costly [20], and has failed 13.2 % of experienced physicians in the last 15 years on their first-time MOC examination [21]. Proponents claim the cost for the program is “the cost of keeping up” [22]. Yet after nearly 30 years of attempting to legitimize the existence of time-limited certification, no credible data exist that the ABMS MOC program has led to improved patient outcomes [23, 24]. Corporate employees and subcontractors without consistent disclosure of conflicts of interest generally author publications in support the ABMS MOC program [25]. Even as late as May 12, 2013, the American Board of Pathology offered ABMS Board re-certification for nothing more than a cash payment of $1000 in lieu of any other requirements, including sitting for a secure examination [26]. In addition, mainstream media sources have identified physicians harmed by the revocation of hospital and insurance privileges after failing their MOC examination [27]. This led the Association of American Physicians and Surgeons to file a lawsuit on behalf of the national membership against the ABMS in April of 2013 seeking redress on multiple issues regarding conspiracy and restraint of trade (see United States District Court for the District of New Jersey Docket No. 3:13-cv-2609-PGS-LHG). The case was moved to Chicago and is currently waiting judgment on a motion to dismiss filed by the ABMS before the Federal District Court in Chicago (See United States District Court for the Northern District of Illinois Docket No. 1:14-CV-2705) at the time of this writing. If the ABMS, a private, insulated non-profit corporation granted tax exempt status by the public to assure accountability of practicing physicians, then practicing physicians and the public have every right to demand the same from the ABMS and its member boards.

Other “accountable” professions (like airline pilots and lawyers) do not require a “maintenance of certification” program to assure public safety or welfare. In the case of pilots, after being granted a flight certificate by the Federal Aviation Administration, with the exception of student and some temporary issue certifications, pilot certificates do not expire, although they may be suspended or revoked [28]. However, pilots are required to maintain their respective ratings by passing rigorous flight reviews with a qualified instructor every 2 years. Likewise, lawyers certified by the American Bar Association in specialized fields of law only need to document their experience in their respective field, provide peer letters, and self-report 30 hours of continuing legal education (CLE) credits and in most states, CLE participation is self-reported [29].

## The ABIM Foundation and the $2.3 million condominium

While the ABIM, a member board of the ABMS, consistently stresses the importance of physician accountability with the public, there is a strong case to be made that the ABIM has not been accountable to its physician members and the public. The ABIM receives 98 % of its revenues from physician testing fees [30]. On October 17, 1989 and just before implementation of time-limited certification, the ABIM quietly registered the “ABIM Foundation” corporation in Pennsylvania [31], and the “American Board of Internal Medicine Foundation” was granted non-profit status with the Internal Revenue Service (IRS) in 1990 [32]. From 1989 to 1999, monetary transfers were made from the ABIM to the “American Board of Internal Medicine Foundation.” To the best of our knowledge, no public disclosure of these monetary transfers ever occurred to the practicing physician community or ABIM diplomats. By June 30, 1999, the “American Board of Internal Medicine Foundation” had accrued over $59.6 million in assets from physician testing fees [33]. Only after an ABIM-requested IRS administrative name change from the “American Board of Internal Medicine Foundation” to the “ABIM Foundation” on March 26, 1999 [34] was this additional ABIM organization, the “ABIM Foundation,” disclosed publicly. In fact, until as late as November 27, 2014, the ABIM Foundation “About Us” webpage still indicated the organization was created in 1999 [35]. Additionally, ABIM Foundation IRS Form 990 tax forms filed from 2008 through 2012 indicated only that organization was formed in 1999 in Iowa (like the ABIM), not in Pennsylvania as disclosed in public record, further obfuscating the origin of the Foundation.

With its prolific balance sheet obtained from practicing physician diplomats and for clinical reasons that remain unclear, the ABIM Foundation leadership felt compelled to organize a “Task Force” to define and promote the concept of “medical professionalism” in 1999. This definition was ultimately published simultaneously as a white paper in the *Annals of Internal Medicine* and *The Lancet* and included a cost-saving “social justice” imperative for physicians [36, 37]. The ABIM continued to issue grants from 2000 to 2007 totaling $20.66 million to their Foundation [38] culminating in the purchase of a $2.3 million luxury condominium complete with a chauffeur-driven Mercedes S-class town car by the Foundation in December 2007 [39]. According to tax records, exclusive meeting venues for the ABIM included such locations as the Ritz-Carlton, Laguna Niguel in California and the Four Seasons Hotel, Philadelphia. Ironically, in 2011, the ABIM Foundation’s “Choosing Wisely©” campaign was launched to promote cost savings in health care. With this program, the Foundation partners with some 70 societies and some non-physician organizations, including Consumer Reports. As part of the campaign, the ABIM Foundation awarded monetary grants sponsored, in part, by the Robert Wood Johnson Foundation and physician fees to third parties willing to “educate practicing physicians.” Meanwhile, by June 30, 2014, the ABIM’s own IRS Form 990 balance sheet showed its net assets or fund balances had dwindled to *negative* $47,886,854 (Line 22), balanced by a promise of $94,075,214 (page 12 of 2013 Form 990) in “deferred income.” [40]

Where, then, is the “deferred revenue” for the ABIM to materialize from? It appears the promise to repay the ABIM comes from physician testing fees in future years and the cementing of the ABMS as a physician quality registry and its MOC program into the Affordable Care Act [41]. To entice physician enrollment in the ABMS MOC program, taxpayer-funded physician payment incentives were issued from 2011 to 2014 by the Center for Medicare and Medicaid Services [42]. Tax disclosures suggest the ABIM had long-term undisclosed lobbying efforts with Congress from at least 2009 to 2014 as confirmed by the Center for Responsive Politics [43]. The extent of government agency lobbying to financially benefit the ABIM and ABMS are unknown. But after disclosure of this lobbying relationship by mainstream media [44] and a physician’s personal weblog [43], the ABIM’s long-standing relationship with its lobbying firm was terminated June 30, 2015 [45]. Ironically, despite these many deceptive practices, the ABIM felt compelled to sanction 139 practicing physicians for “ethical violations” in June of 2010 because of threats to their intellectual property [46].

## The early corrections

Fortunately, the public disclosure of these facts has already created a much-needed correction to the unproven ABMS MOC re-certification program conducted by the ABIM, both within the organization and from its subsidiary subspecialty boards and specialty medical societies.

Within the ABIM, numerous steps were taken to “transform” MOC from its 2014 iteration. First, there was a public apology that “we got it wrong” [47]. Conventional CME credits were allowed to “count” for MOC points, website reporting will change to “participating in MOC” rather than “meeting MOC requirements,” a 2-year suspension of the Practice Assessment, “Patient Voice,” and Patient Safety requirements for the MOC program were made and the fees for the program capped through 2017. Unfortunately, none of these addressed the public concerns of the financial actions of the organization.

## The specialty society response

In response to the current ethical, procedural, and financial practices of the ABIM, a new board, National Board of Physicians and Surgeons (NBPAS), spearheaded by Paul Teirstein, MD, was launched as an alternative pathway for re-certification purposes [48]. Only a first ABIM certification and proof of 50 hours of credible CME within the prior 24 months are required to “maintain” certification with NBPAS. So far, 18 hospitals have agreed to accept this re-certification credential [48]. The costs for re-certification are a fraction of the ABMS MOC program and at the present time Board members of the NBPAS are unpaid.

Other professional societies are moving to modify the ABMS MOC program or alter it completely. The American Board of Anesthesiology is proposing completion of a trademarked “MOCA Minute™” interactive “learning tool” study questions weekly in lieu of performing the “MOCA Part 3 secure examination” [49]. The American Gastroenterological Society is petitioning the ABIM to remove the MOC requirement for gastroenterologists in favor of their Gastroenterologist: Accountable Professionalism in Practice (G-APP) program [50]. While other subspecialty boards are likely to follow, the cost of participation for these unproven programs, the many conflicts of interest that exist with the ABMS Specialty Boards, and the true value of re-certification to physicians and patient care remain unproven.

## Conclusions

The level of vetting for established physicians to earn their medical degree and initial subspecialty certification is extensive and the sum of the entities scrutinizing their individual behavior is comprehensive and redundant: State Medical Boards, the Drug Enforcement Agency, hospital Medical Executive Boards, the Center for Medicare and Medicaid Services billing data, third party payers, individual subspecialty organizations, county medical organizations and the like. Couple this with the additional levels of scrutiny from mainstream media outlets, “Big Data” access, claims-based data analysis, Google searches, and Yelp reviews available via a smartphone or a laptop. This mix of data and profiling may serve to keep the medical field open and honest at least as well as a series of expensive multiple-choice examinations.

So the questions become these: if the federal, state, and local governments, hospitals, individual subspecialty societies, lay press, social media, tort system, and medical insurance companies all direct physician behavior, what exactly is the ABIM trying to regulate that isn’t already being regulated? Where is the evidence that the costly and time-consuming re-certification system improves patient care?

If one asks the question, “should the ABIM (or any ABMS member board) be the sole arbiter and regulator of ‘re-certification’” when many of us recognize that this role is already filled and the answer is a resounding “no,” then this debate is over. The ABMS and their member boards have lost their way, especially in regards to the costs and proof of the value of their MOC re-certification program. They have also lost the trust of the practicing physician community. While it is encouraging to practicing physicians that changes are underway, time will tell if the lucrative Specialty Board system in the USA can withstand the significant ongoing legal challenges and adapt to a more transparent, economic, and practical system that returns to serving the needs to today’s practicing physician community and their patients, rather than themselves. It is fair to say that significant financial conflicts of interest existed for the implementation of time-limited specialty certification and these continue to plague the U.S.-based ABMS Specialty Board system’s credibility. To rectify the situation and restore trust among practicing physicians and their patients, the specialty boards and the organizations that created them should remove all requirements for time-limited board certification and resort to conventional self-selected ACGME-approved CME programs for ongoing professional education.
